# Identification of Key Genes Related to Intramuscular Fat Content of Psoas Major Muscle in Saba Pigs by Integrating Bioinformatics and Machine Learning Based on Transcriptome Data

**DOI:** 10.3390/ani15081181

**Published:** 2025-04-20

**Authors:** Zixia Huang, Yongli Yang, Jinhua Lai, Qiang Chen, Xiaoyi Wang, Shuyan Wang, Mingli Li, Shaoxiong Lu

**Affiliations:** Faculty of Animal Science and Technology, Yunnan Agricultural University, Kunming 650201, China; 13286171928@126.com (Z.H.); 15987169785@163.com (Y.Y.); jinhual24@163.com (J.L.); chq@sjtu.edu.cn (Q.C.); wangxiaoyi0101@126.com (X.W.); shuyanwang2014@126.com (S.W.); xiaolucao@126.com (M.L.)

**Keywords:** Saba pig, psoas major muscle, intramuscular fat content, integrated bioinformatics, machine learning, hub gene, pork

## Abstract

The psoas major muscle (PMM) is a piece of pork with good tenderness and high value. Intramuscular fat (IMF) content, serving as a pivotal indicator of pork quality, varies greatly among pigs within the same breed. However, there is a paucity of studies focusing on investigating the molecular mechanism of PMM IMF deposition in the same pig breed. The present study aimed to identify the potential genes related to the IMF content of PMM in low- and high-IMF Saba pigs based on transcriptome data analysis. The data used in this study were the RNA sequences of PMM from 12 Saba pigs (PRJNA1223630, from our laboratory) and gene expression profiles (GSE207279) acquired from the NCBI Sequence Read Archive database and the GEO database, respectively, together with data on the fatty acid and amino acid composition of the 12 Saba pigs’ PMM. It was found that the high-IMF pigs exhibited significantly elevated levels of saturated fatty acids and (mono)unsaturated fatty acids, especially C14:0, C16:0, C20:0, C16:1, C18:1n9c, and C20:2, compared with those in the low-IMF pigs (*p* < 0.05 or *p* < 0.01). A total of 370 differentially expressed genes (DEGs) (221 up- and 149 down-regulated) were identified based on PRJNA1223630. Then, 20 hub genes were identified through protein–protein interaction (PPI) network analysis. Four potential fat-deposition-related genes (*DGAT2*, *PCK1*, *MELK*, and *FASN*) were further screened via the intersection of the candidate genes identified by the Least Absolute Shrinkage and Selection Operator (LASSO) algorithm and the top five genes ranked by the Random Forest (RF) method based on the 20 hub genes and were validated in the test gene set (GSE207279). The constructed mRNA (gene)–miRNA–lncRNA network, involving miRNAs (miR-103a-3p, miR-107, and miR-485-5p), lncRNAs (XIST, NEAT1, and KCNQ1OT1), and *FASN*, might be crucial for IMF deposition in pigs. These findings might delineate valuable regulatory molecular mechanisms coordinating IMF deposition and could serve as a beneficial foundation for the genetic improvement of pork quality.

## Simple Summary

Elucidating the regulatory factors that affect intramuscular fat (IMF) is essential for pork quality enhancement. While the psoas major muscle (PMM), as a prototypical oxidative muscle fiber, demonstrates exceptional tenderness and favorable sensory attributes, the molecular mechanisms governing IMF content and meat quality divergence remain to be exhaustively detailed. We conducted a systematic comparative transcriptomic analysis of the PMM between low- and high-IMF indigenous Chinese Saba pigs to investigate PMM IMF-specific differences in meat quality. We found that the high-IMF pigs exhibited significantly elevated saturated fatty acid and (mono)unsaturated fatty acid content. We also identified key genes governing adipose tissue accumulation in PMM and influencing meat quality that regulate fat deposition in PMM and overall pork quality. Our findings delineate key biomarkers and develop molecular regulatory networks associated with fat metabolism in skeletal muscle, thereby establishing a foundation for a comprehensive investigation into the molecular mechanisms that govern pork IMF deposition.

## 1. Introduction

Intramuscular fat (IMF) content is a critical determinant of pork quality, exhibiting a positive correlation with palatability, marbling score, tenderness, juiciness, and meat characteristics [1]. It is widely recognized that an elevated IMF level serves as a reliable indicator of high-quality pork [2]. In pigs, IMF demonstrates a relatively high degree of heritability, with estimated values ranging from 0.21 to 0.86 and an approximate mean of 0.5 [3,4]. This significant heritability renders porcine IMF a valuable criterion for selection in breeding programs aimed at improving pork quality and carcass fatness [5]. The evolution of next-generation sequencing technologies has facilitated the widespread adoption of molecular breeding in enhancing meat quality in livestock and poultry. Consequently, molecular breeding strategies are poised to offer a more effective and sustainable approach for improving the IMF content in swine. Moreover, the identification of potential molecular markers for IMF has emerged as a critical objective within genetic research and pig molecular breeding programs. For example, genes such as *AGT*, *EMG1*, and *PCTP* were identified as candidate genes associated with IMF in the crossbred offspring of Luchuan sows and Duroc boars [6]. Furthermore, pigs serve as an exemplary model for investigating human-obesity-related issues, given their physiological and genomic similarities to humans [7]. Therefore, research into the molecular mechanisms underlying IMF deposition is essential for advancing both the economic viability of pig production and human health outcomes.

Transcriptomic analyses that compare individuals exhibiting polarized phenotypes of specific characteristics are essential for elucidating molecular interaction relationships that demonstrate divergent levels of expression across various pig breeds. It is well known that different pig breeds exhibit significant variations in their meat quality traits. The IMF content of different pig breeds varies significantly, ranging from 2% to 10% [8,9]. For instance, indigenous Chinese pig breeds (such as Laiwu, Jinhua, Meishan, and Wei pigs) typically exhibit higher IMF levels compared with their Western counterparts and commercial breeds (such as Landrace, Large White, and Duroc pigs), which contributes to superior meat quality [10,11]. Nevertheless, there are few investigations focusing on individuals of the same breed that possess differential IMF levels. Elucidating the molecular mechanisms governing IMF accumulation is essential for enhancing the productivity and availability of high-quality pig production [12]. Many factors influence IMF content, such as breed, diet, and feeding practices. Notably, individuals of the same breed often display significant variability in IMF levels, even when raised in the same environment. Consequently, compared with pigs across different breeds, pigs exhibiting divergent IMF contents within the same breed might be ideal for screening potential biomarkers that influence IMF deposition, which would mitigate the effects of the genetic background across distinct breeds. The Saba pig is a prized indigenous Chinese pig breed that is predominantly distributed in the central region of Yunnan Province. It is well established that Saba pigs demonstrate superior meat quality with high IMF in the longissimus dorsi muscle (LDM) (>6%) and have a coefficient of variation of over 25% in IMF content [13], which make them ideal animal models for studying the genetic mechanism of IMF traits.

In addition, the muscle structure and its properties are intricately linked to the types of muscle fibers, which in turn have a significant impact on various meat quality attributes, such as water-holding capacity and tenderness [14]. As we know, the LDM is a representative muscle in a pig’s carcass, as it is the reference muscle and commonly used in meat-quality-related studies. The psoas major muscle (PMM) is an oxidative muscle with a high proportion of Type I and IIa fibers that exhibits superior tenderness and sensory qualities [15,16,17]. However, research investigating the molecular mechanisms underlying the differences in fat deposition and meat quality between PMMs with low and high IMF remains limited to those of the LDM. The study of the meat quality differences between low- and high-IMF content in the PMM not only complements the differences in meat quality among different muscle types in pigs but also further identifies the main molecular mechanisms that affect the quality of premium pork parts.

In general, IMF deposition is a significant factor that contributes to pork quality, and addressing the molecular mechanisms may offer new prospects for improving meat quality in pig breeding. The present research implemented an integrated bioinformatics and machine learning strategy to identify the potential genes linked to IMF content and the meat quality of PMM in Saba pigs that exhibited varying levels of IMF content based on transcriptome data. Machine learning algorithms are capable of effectively analyzing high-dimensional data and exhibit a substantial amount of potential in the identification of biomarkers associated with specific traits [18]. Additionally, this approach has increasingly been utilized to evaluate economically significant traits in livestock, such as growth [19] and meat quality characteristics [20]. Additionally, we aimed to develop molecular regulatory networks associated with these specific genes, thereby establishing a foundation for a comprehensive investigation into the molecular mechanisms that govern pork IMF deposition.

## 2. Materials and Methods

### 2.1. Data Collection and Preprocessing

The data used in this study were from 12 Saba pigs (six males and six females) and included RNA sequences (NCBI Sequence Read Archive database: PRJNA1223630, submitted by our laboratory) and physiochemical indices comprising the fatty acid and amino acid composition of PMM. The 12 pigs were selected from 30 IMF-tested Saba pigs (15 males and 15 females). At the age of 320 days and weighing approximately 100 kg, the 30 pigs were slaughtered, and the samples of PMM of each animal were collected immediately for transcriptome sequencing and IMF content determination. Twelve samples were allocated into low-IMF and high-IMF groups, with six samples in each group (three males and three females), which were selected from the animals with the lowest and highest IMF content, respectively, of the 30 IMF-tested pigs. Detailed information about the 12 animals in this study is shown in Table 1. Additionally, the dataset GSE207279 sourced from the GEO database (https://www.ncbi.nlm.nih.gov/geo/ (accessed on 12 September 2024)) was employed for validation purposes, comprising six samples (three each of the high- and low-IMF pigs).

### 2.2. Measurement of Fatty Acid and Amino Acid Composition in PMM

The selected 12 PMM samples of Saba pigs were further analyzed for their fatty acid and amino acid composition. The composition of fatty acids in PMM was measured using gas chromatography, adhering to the Determination of Fatty Acids in Food Products standard (GB 5009.168-2016). The amino acid composition was determined using an ultra-high-speed automatic amino acid analyzer (Hitachi, Tokyo, Japan), following the method described in the Chinese National Food Safety Standard (GB 5009.124-2016) for the Determination of Amino Acids in Food. For detailed methods, see the article of Ge et al. [21].

### 2.3. Detection of Differentially Expressed Genes (DEGs)

Differential expression analysis comparing the low- and high-IMF groups was conducted with the “DESeq2” package in R (version 4.3.2) [22]. Genes meeting the threshold criteria (|log_2_FC (fold change)| > 1; *p*-value < 0.05) were considered differentially expressed. Their expression patterns are graphically represented through heatmap and volcano plot visualizations constructed with ggplot2 (version 3.5.0).

### 2.4. Functional Enrichment Analysis of DEGs

Functional characterization of DEGs was implemented via DAVID (2021, https://david.ncifcrf.gov/home.jsp (accessed on 15 October 2024)) for GO and KEGG pathway analyses [23]. The GO terms were classified into three distinct domains: cellular component (CC), biological process (BP), and molecular function (MF). Significantly enriched biological annotations (*p* < 0.05) were identified, and these terms are graphically represented through visualizations constructed with the ggplot2 package in R (version 3.5.0).

### 2.5. Protein–Protein Interaction (PPI) Network Analysis and Hub Gene Recognition

The relationships among proteins encoded by DEGs were examined through the construction of a PPI network via the STRING online database (https://cn.string-db.org/ (accessed on 20 October 2024)), implementing a combined score threshold of >0.4 and a *p*-value of <0.05 [24]. It was visualized in Cytoscape (version 3.8.0) with nodes representing gene-encoded proteins and the edges indicating their interactions [25]. Subsequently, a cluster analysis of the entire PPI network was conducted utilizing the Molecular Complex Detection (MCODE) algorithm (version 2.0.2) [26]. This analysis utilized the following parameter configurations: a degree threshold of 2, a node scoring threshold of 0.2, a k-value of 2, and the exploration depth constrained to 100 levels. The identification of hub genes was performed based on the Maximal Clique Centrality (MCC) algorithm via the Cytoscape plugin cytoHubba (version 0.1) [27], resulting in the selection of the MCC-ranked gene cohort (*n* = 20) that emerged as central regulatory nodes governing modular connectivity, that is, the hub genes. The functional associations among hub genes with GO annotations and KEGG pathway enrichments were mapped through ClueGo (version 2.5.9) and the CluePedia plugin (version 1.5.9) [28,29].

### 2.6. Screening of Potential IMF-Related Genes

The Least Absolute Shrinkage and Selection Operator (LASSO) [30] and the Random Forest (RF) algorithm [31] were implemented to further screen potential IMF feature-related genes. Initially, the “glmnet” R package was deployed to conduct the LASSO analysis [32]. Concurrently, the “randomForest” R package was employed for the RF analysis [33]. Then, the intersection of two sets of potential fat-deposition-related genes was taken to obtain the optimal potential genes. To assess the predictive performance of the identified genes, we analyzed their expression level differences across the low- and high-IMF pigs through an unpaired *t*-test, where a *p*-value less than 0.05 was deemed statistically significant. Subsequently, receiver operating characteristic (ROC) curves were implemented, and the area under the curve (AUC) values for the potential genes were calculated based on the validation dataset GSE207279 in order to evaluate the distinguishing efficacy of potential IMF-related genes, utilizing GraphPad Prism (version 10.2.3).

### 2.7. Gene Set Enrichment Analysis of Potential IMF-Related Genes

To further explore the potential IMF-related genes’ capabilities, gene set enrichment analysis (GSEA) was conducted through the “clusterProfiler” (version 3.4.4) and “GSEABase” (version 1.38.1) packages in R [34], and significantly enriched terms were plotted with the “enrichplot” package (version 3.5.1). To enhance our comprehension of the roles of the potential genes, a correlation investigation involving hallmark gene sets was conducted utilizing single-sample gene set enrichment analysis (ssGSEA) [35]. The ssGSEA scores for the hallmark gene set across both the low- and high-MF cohorts were calculated. Then, the relationship between the hallmark gene sets and the potential genes were investigated, employing the “corrplot” (version 0.84) package to derive the Spearman rank correlation coefficient.

### 2.8. Construction of the mRNA (Gene)–miRNA–lncRNA Regulation Network

The interactive relationships among miRNAs, lncRNAs, and mRNAs were elucidated through the construction of a regulatory network. Potentially targeted miRNAs and lncRNAs associated with prospective genes were forecasted using the online ENCORI database (https://rna.sysu.edu.cn/encori/index.php (accessed on 25 January 2025)). Initially, gene–miRNA regulatory pairs were mined from a minimum of two databases [36], which included miRanda [37], TargetScan [38], PITA [39], and miRmap [40]. Subsequently, the lncRNAs were inferred using the previously identified miRNAs. Then, the coexpression network of miRNAs, lncRNAs, and mRNAs (candidate genes) within the regulation network was constructed via Cytoscape software (version 3.8.0).

### 2.9. Verification of RNA Sequencing Results by RT-qPCR

The experimental samples utilized in RT-qPCR were obtained from prior research conducted in our laboratory [20]. Based on the variations in the IMF content of PMM, samples from Saba pigs exhibiting both low and high levels of IMF in PMM were chosen to characterize the expression signatures of the identified potential genes. Whole RNA was isolated from PMM tissues via the RNA Sample Total Extraction Kit (Tiangen, Beijing, China), followed by gDNA Eraser-integrated reverse transcription with the PrimeScript™ RT Reagent Kit (Takara, Dalian, China) per the manufacturer’s protocols. RT-qPCR assays were executed utilizing TB Green^®^ Premix Ex Taq™ II (Tli RNaseH Plus) (Takara, Dalian, China) on an Mx3000P qPCR system (Agilent Technologies, Santa Clara, CA, USA). The primers specific to the genes analyzed in the RT-qPCR are detailed in Supplementary Table S1. Each experimental condition was replicated three times, and the relative mRNA expression levels were quantified using the 2^−ΔΔCt^ approach normalized against *GAPDH* as the endogenous reference.

### 2.10. Statistical Analysis

Meat quality parameters and relative mRNA expression levels were statistically compared between the low- and high-IMF groups through an unpaired *t*-test, following normality verification by the Shapiro–Wilk method and a homogeneity of variance assessment by Levene’s test. All statistical analyses were performed using SAS 9.2. The results of meat quality parameters are presented as the mean ± standard deviation, with *p*-values < 0.05 and *p*-values < 0.01 indicating statistically significant and exceptionally significant results, respectively.

## 3. Results

### 3.1. The Fatty Acid and Amino Acid Composition of PMM Samples

As shown in Table 2, the fatty acid profiles showed no statistically significant differences in the content of ΣPUFA, Σω-3, and Σω-6 in the low- and high-IMF pigs (*p* > 0.05). In contrast, the low-IMF group exhibited markedly reduced concentrations of C14:0, C16:0, and C20:0 compared with the high-IMF cohort (*p* < 0.05). Conversely, the high-IMF cohort displayed notably elevated levels of C16:1, C18:1n9c, and C20:2 (*p* < 0.05). Additionally, the high-IMF group exhibited significantly elevated levels of ΣSFA (*p* < 0.05), ΣUFA (*p* < 0.05), and ΣMUFA (*p* < 0.01). It is noteworthy that, although no statistically significant variations were found in the compositional profiles of amino acids, the overall amino acid content was at a superior level, with the EAA/TAA and EAA/NEAA ratios exceeding 40% and 60%, respectively (Table 3).

### 3.2. DEG Identification

**Figure 1:**
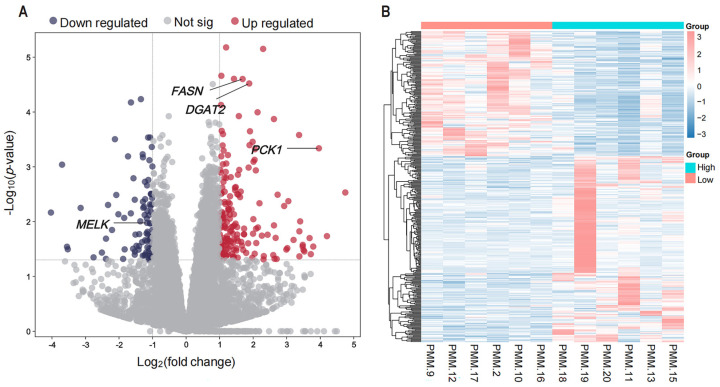
DEG identification. (**A**) The volcano plot of DEGs Each dot represents a gene, with red and bluish dots indicating up–regulated and down–regulated genes, respectively. (**B**) The cluster heatmap of DEGs.

A total of 370 DEGs (221 up- and 149 down-regulated) were detected in the low- and high-IMF pigs (*p* < 0.05) (Figure 1A, Supplementary Table S2). Transcript levels for the identified DEGs across the low- and high-IMF pigs are depicted in the heatmap presented in Figure 1B.

### 3.3. Functional Enrichment Analysis of DEGs

**Figure 2:**
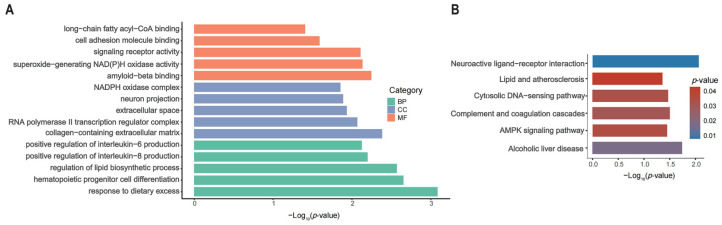
DEG functional analysis. (**A**) DEG GO analysis results. (**B**) DEG KEGG analysis results.

GO analysis of the DEGs revealed significant enrichment in 20 BPs, seven CCs, and eight MFs (*p* < 0.05) (Supplementary Table S3). The five most enriched GO terms in *p*-values are illustrated in Figure 2A. Among the enriched BPs, DEGs showed significant enrichment in functions related to the response to dietary excess, hematopoietic progenitor cell differentiation, and regulation of the lipid biosynthetic process. The CCs were mainly involved the collagen-containing extracellular matrix, RNA polymerase II transcription regulator complex, and NADPH oxidase complex. The MFs primarily dealt with superoxide-generating NAD(P)H oxidase activity, signaling receptor activity, and long-chain fatty acyl–CoA binding. Additionally, KEGG enrichment analysis of the DEGs identified six pathways that were significantly enriched (*p* < 0.05) (Supplementary Table S3), including neuroactive ligand–receptor interaction, alcoholic liver disease, complement and coagulation cascades, the cytosolic DNA-sensing pathway, lipid and atherosclerosis, and the AMPK signaling pathway, as depicted in Figure 2B.

### 3.4. PPI Network Construction

**Figure 3:**
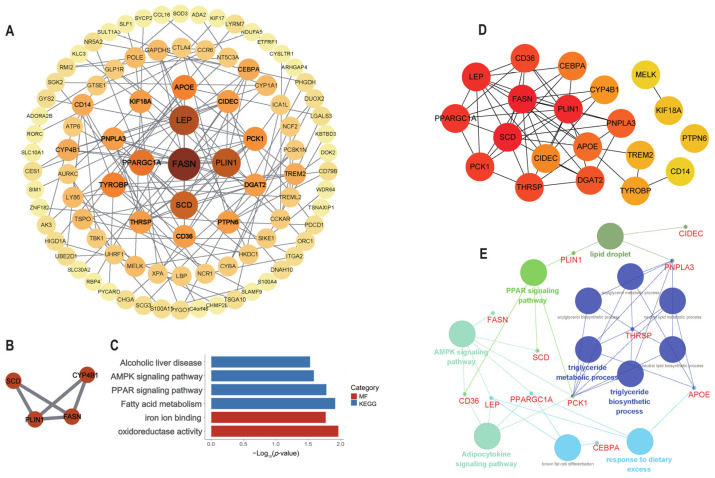
Hub gene determination and functional characterization. (**A**) PPI network with 158 nodes and 198 edges based on 370 DEGs. (**B**) Top-scoring PPI network module. (**C**) Significant functional enrichment outcomes of the cluster genes. (**D**) Twenty hub genes were identified. (**E**) Noteworthy functional enrichment outcomes of the 20 hub genes.

Totals of 158 nodes and 198 edges were identified (Figure 3A) by PPI analysis of the 370 DEGs. In the modules identified by MCODE, the strongly associated cluster with the highest ranking (score = 3.333) was selected, which comprised four nodes and five edges (Figure 3B). The enrichment analysis results indicate that the genes within this module were predominantly linked to fat metabolism, such as the AMPK and PPAR signaling pathways, and fatty acid metabolism (Figure 3C).

### 3.5. Identification and Analysis of Hub Genes

Within the comprehensive analysis of the PPI network, the top 20 genes exhibiting the highest MCC scores were identified as hub genes (*FASN*, *PLIN1*, *SCD*, *LEP*, *PPARGC1A*, *APOE*, *THRSP*, *CD36*, *DGAT2*, *PCK1*, *CIDEC*, *PNPLA3*, *CEBPA*, *CYP4B1*, *TYROBP*, *TREM2*, *CD14*, *KIF18A*, *MELK*, and *PTPN6*; Figure 3D). There were 16 hub genes that exhibited upregulation in the high-IMF group, except for *PPARGC1A*, *CD36*, *KIF18A*, and *MELK*. The interactive relationships among these 20 hub genes, along with the associated GO and KEGG terms, are depicted in Figure 3E. The fat-metabolism-related pathways were enriched, such as the AMPK signaling pathway (*FASN*, *SCD*, *PCK1*, *PPARGC1A*, *LEP*, *CD36*), the PPAR signaling pathway (*PLIN1*, *PCK1*, *SCD*, *CD36*), and the adipocytokine signaling pathway (*CD36*, *LEP*, *PPARGC1A*, *PCK1*).

### 3.6. Identification of Potential IMF-Related Genes

**Figure 4:**
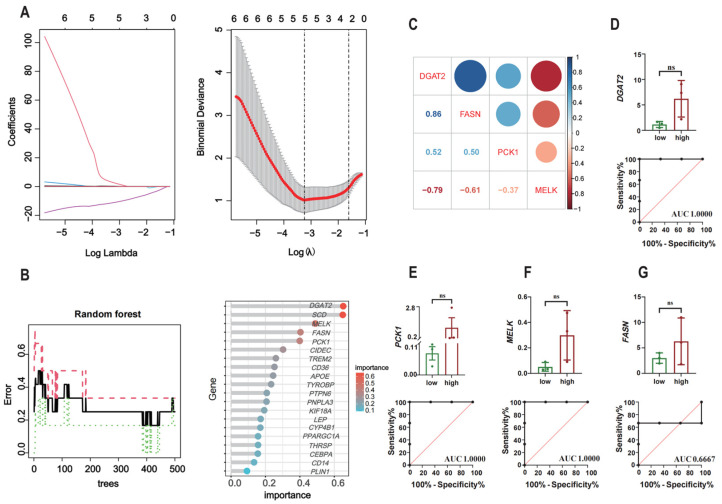
Identification of potential signature genes. (**A**,**B**) The LASSO and RF methods were used to filter out potential genes sequentially. (**C**) Correlations among potential genes. (**D**–**G**) Barplot depicting the expression of genes comparing the low- and high-IMF groups in the validation cohort, accompanied by ROC curves evaluating the predictive capacity of (**D**) *DGAT2*, (**E**) *PCK1*, (**F**) *MELK*, and (**G**) *FASN*. ns: no significant difference.

**Figure 5:**
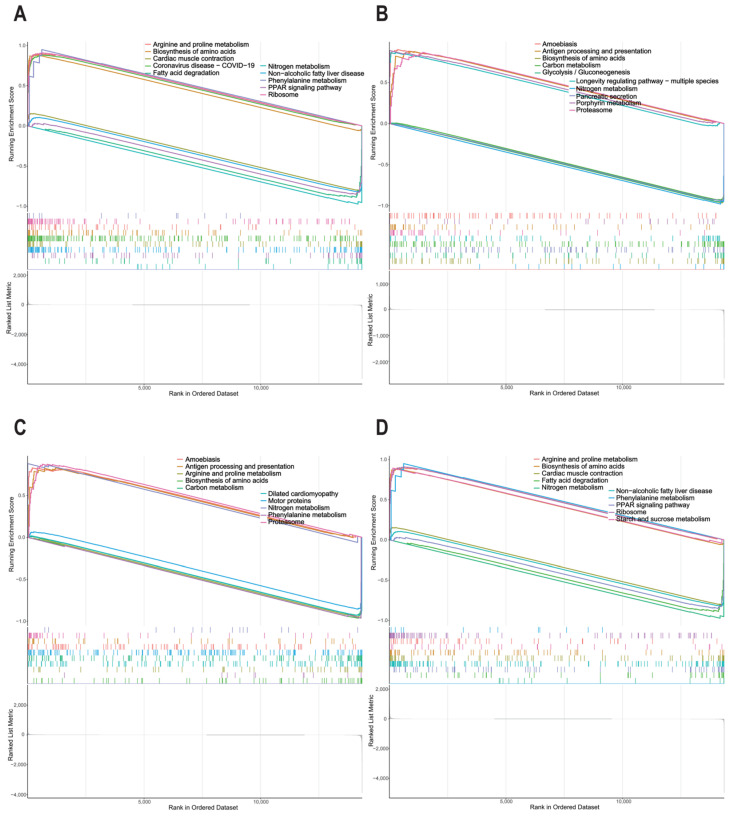
Functional analysis of candidate obesity-specific genes by GSEA. (**A**) *DGAT2*. (**B**) *PCK1*. (**C**) *MELK*. (**D**) *FASN*.

Based on the 20 hub genes, five candidate genes were screened through the LASSO regression algorithm (Figure 4A). Additionally, the 20 genes were ranked according to their calculated importance by the RF algorithm (Figure 4B). The intersection of the candidate genes identified by LASSO and the top five genes ranked by the RF algorithm led to the identification of four genes, *DGAT2*, *PCK1*, *MELK*, and *FASN*, as potential feature genes associated with IMF deposition. With regard to the level of gene expression, *DGAT2* had a strong positive correlation with *FASN* (Figure 4C). The results of the ROC analysis demonstrate that the AUC values of *DGAT2*, *PCK1*, *MELK*, and *FASN* were 1, 1, 1, and 0.6667, respectively (Figure 4D–G), indicating that the four genes also have excellent specificity and sensitivity, making them potential signature genes in PMM with varying levels of IMF content. Furthermore, the GSEA results indicate that pathways associated with amino acid metabolism, such as the biosynthesis of amino acids and nitrogen metabolism, were notably enriched by these four genes. Arginine and proline metabolism and phenylalanine metabolism were significantly enriched by *DGAT2*, *MELK*, and *FASN*. Additionally, pathways associated with fat metabolism, including fatty acid degradation and the PPAR signaling pathway, were notably enriched by *DGAT2* and *FASN* (Figure 5A–D).

### 3.7. Analysis of Hallmark Gene Sets of Potential IMF-Related Genes

**Figure 6:**
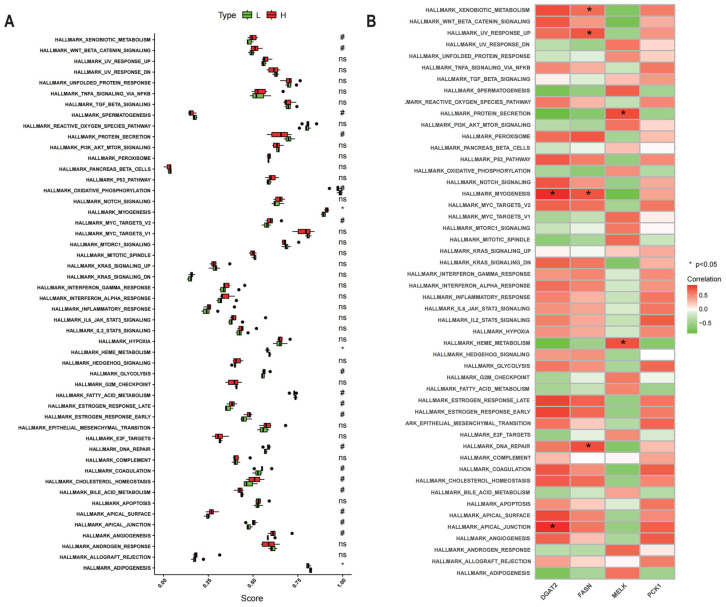
Association analysis of candidate genes with the hallmark gene sets across the low- and high-IMF cohorts. (**A**) ssGSEA activity levels of hallmark gene sets and transcriptomic signatures. (**B**) Link between hallmark gene sets and possible genes. *: *p* < 0.05, significant difference. #: *p* < 0.2, no significant difference. ns. *p* < 1 no significant difference.

Hallmark myogenesis in the high-IMF pigs was discovered to be significantly elevated in comparison with the low-IMF pigs, while the hallmark pathways associated with heme metabolism and adipogenesis were notably diminished (Figure 6A). In terms of potential genes (Figure 6B), *DGAT2* exhibited a positive correlation with both myogenesis and apical junction integrity (*p* < 0.05). Additionally, *FASN* demonstrated a significant association with myogenesis as well as DNA repair mechanisms (*p* < 0.05). *MELK* was significantly linked to protein secretion and heme metabolism (*p* < 0.05). *PCK1* did not exhibit a significant link with the hallmark gene sets (*p* > 0.05).

### 3.8. Development of the mRNA (Gene)–miRNA–lncRNA Interaction Network

**Figure 7:**
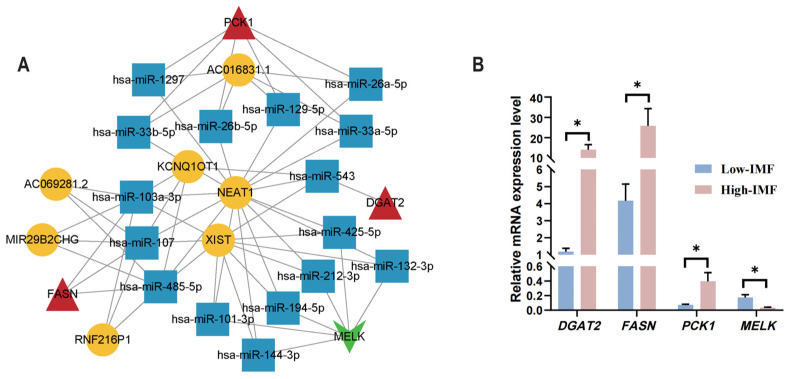
Construction of the mRNA–miRNA–lncRNA network and validation of the mRNA expression levels of the potential genes. (**A**) mRNA–miRNA–lncRNA interaction network comprised of four candidate genes, 16 miRNAs, and seven lncRNAs. Genes are featured in red triangles (up-regulated genes) and green arrows (down-regulated genes), whereas targeted miRNAs and lncRNAs are displayed in blue squares and yellow circles, respectively. (**B**) Confirming mRNA levels in candidate genes via RT-qPCR. The symbol * means *p* < 0.05.

In the context of the latent signature genes associated with IMF deposition (*DGAT2*, *PCK1*, *MELK*, and *FASN*), a total of seven lncRNAs were predicted from 16 miRNAs corresponding to these four genes. This analysis led to the establishment of 45 lncRNA–miRNA interactions and 16 miRNA–mRNA interactions, as illustrated in Figure 7A. The predictive outcomes regarding the miRNAs and lncRNAs associated with these four genes suggest that the lncRNAs KCNQ1OT1, NEAT1, and XIST may function as sponges for various miRNAs, thereby modulating the expression of genes related to IMF deposition.

### 3.9. Validation of the Potential Genes via RT-qPCR

The expression patterns of the four potential feature genes, *DGAT2*, *PCK1*, *FASN*, and *MELK*, in PMM samples were consistent with the findings from the transcriptomic profile, thereby strengthening their reliability. Furthermore, notable differences in the transcript abundance of these four genes were detected across the low- and high-IMF groups (Figure 7B).

## 4. Discussion

Meat quality is a multifactorial trait influenced by diverse factors, among which the IMF content in skeletal muscle has a significant impact. In this study, we investigated the variations in fatty acid and amino acid profiles of low- and high-IMF PMM in Saba pigs. The fatty acids serve as a primary source of flavor in pork, with certain types acting as essential precursors to various flavor compounds that contribute to the overall sensory experience of meat [41]. Research indicates that PUFA levels are inversely linked to pork quality traits, whereas the levels of SFA and MUFA exhibit a direct positive correlation with meat quality [42,43]. Elevated SFA and MUFA levels are associated with enhanced acceptability in terms of flavor, juiciness, tenderness, and overall quality, thereby increasing the palatability of pork [44]. The degree of adiposity influences the fatty acid composition of meat, as an increase in fatness leads to a more rapid rise in SFAs and MUFAs. This change corresponds with a reduction in the relative proportion of PUFAs and a decrease in the PUFA/SFA ratio [45]. We found that high-IMF Saba pigs exhibited significantly elevated SFA, UFA, and MUFA content in PMM compared with the low-IMF cohort, suggesting superior meat quality in the high-IMF pigs, which aligns with the previous research. Therefore, divergent molecular mechanisms likely underlie the metabolic differences in fat and fatty acids across low- and high-IMF PMM.

In this study, comparative transcriptomic analysis of low- vs. high-IMF PMM identified 370 DEGs. Functional analysis of these DEGs indicated a predominant association with processes related to fat deposition, including the regulation of lipid biosynthetic processes [46] and long-chain fatty acyl–CoA binding [47]. After PPI network and machine learning analysis, four robust potential genes (*DGAT2*, *PCK1*, *MELK*, and *FASN*) were screened as IMF-deposition-signature genes.

DGAT2 (diacylglycerol O-acyltransferase 2) is a pivotal enzyme that facilitates the terminal step in triglyceride synthesis [48], which is essential for various physiological processes including intestinal fat absorption, lipoprotein aggregation, regulation of plasma triglyceride levels, adipocyte fat storage, and energy metabolism in muscle tissues [49]. An earlier study demonstrated a positive association between *DGAT2* activity and IMF deposition level among Laiwu, Lulai Black, and Large White pigs [50]. Additionally, a polymorphism within a 13 bp intron in the 3ʹ-UTR of the porcine *DGAT2* gene was found to be linked to backfat deposition and carcass leanness [51]. It was reported that *DGAT2* gene expression level showed a positive association with IMF deposition in Korean beef cattle LDM [52] and with IMF accumulation level and amount of intramuscular adipocytes in breast muscle of domestic pigeons [53]. Furthermore, evidence suggests that the overexpression of *DGAT2* enhances the expression of genes associated with lipid formation and increases the accumulation of triacylglycerols in skeletal muscle cells [54]. This overexpression was also found to significantly upregulate the mRNA levels of *PPARγ*, *C/EBPα*, *C/EBPβ*, *FABP4*, *SREBF1*, and triacylglycerol-synthesis-linked genes such as *GPAT4* and *LPIN1*, thereby regulating adipogenesis [55]. All these findings suggest that *DGAT2* critically influences IMF accumulation.

PCK1 (phosphoenolpyruvate carboxykinase 1) serves as a significant regulator of gluconeogenesis, glyceroneogenesis, and cataplerosis within the tricarboxylic acid (TCA) cycle [56]. *PCK1* facilitates the conversion of oxaloacetate to phosphoenolpyruvate (PEP) utilizing guanosine triphosphate (GTP) while concurrently releasing guanosine-5′-diphosphate (GDP) and CO_2_. This enzymatic activity is integral not only to gluconeogenesis but also contributes to glycerol production [57]. The TCA cycle modulates PEP levels via *PCK1*, thereby influencing gluconeogenesis and lipid synthesis. An increase in *PCK1* expression can enhance the TCA cycle, resulting in elevated production of GDP and succinyl-CoA synthetase, which in turn augments energy metabolism [58]. Studies have identified *PCK1* as a potential adipogenic marker, an obesity-related gene, and a gene implicated in IMF deposition [58,59]. For instance, a marked positive association has been demonstrated between *PCK1* expression and IMF content in Duroc × Shanzhu commercial crossbred pigs [59]. Furthermore, transgenic mice that overexpressed the cytosolic form of *PCK1* in skeletal muscle exhibited nearly a fourfold increase in IMF content [58]. Additionally, *PCK1* expression was upregulated in Wagyu × Hereford crossbred cattle, which were characterized by high IMF content, compared with Piedmontese × Hereford crossbred cattle, which had low IMF content [60]. *PCK1* also demonstrated a positive correlation with the IMF accumulation level in buffalo [61]. We also identified that *PCK1* expression is positively associated with the IMF content in the PMM of Saba pigs, and further substantiated the pivotal regulatory role of *PCK1* in IMF deposition.

MELK (maternal embryonic leucine zipper kinase), also referred to as MPK38 (murine protein serine-threonine kinase 38), is classified within the AMPK2 (AMP-activated protein kinase)-related serine/threonine kinase family [62]. This protein kinase exhibits cell-cycle-dependent activity and is implicated in the modulation of numerous biological processes, including cell proliferation [63], spliceosome assembly [64], hematopoiesis [65], stem cell self-renewal [66], and apoptosis [67]. While the majority of studies concerning *MELK* have focused on these biological functions, there is a paucity of studies addressing its role in adipogenesis. Notably, mice deficient in *MPK38* (*MPK38*^−/−^) exhibit an increased lipid synthesis rate and elevated expression levels of mRNAs that encode lipogenic proteins (such as *FAS*, *SCD1*, and *SREBP1c*) as well as gluconeogenic proteins (including *G6PC*, *PCK1*, and *PGC1β*) compared with their wild-type counterparts [68]. This observation points to the conclusion that *MELK* may represent a viable target for obesity prevention. Our study revealed a marked decrease in *MELK* expression within PMM tissue when comparing pigs with high-IMF levels to those with low-IMF levels. It is worth noting here that *PCK1* was also detected in the high-IMF PMM. In summary, *MELK* and its negative regulatory relationship with *PCK1* may be a critical factor related to IMF content, but the exact mechanisms through which they affect IMF deposition remain to be fully elucidated.

FASN (fatty acid synthase) is recognized as a crucial metabolic enzyme that significantly influences the composition and accumulation of body fat in mammals. It is integral to lipogenesis, facilitating the synthesis of saturated long-chain fatty acids from acetyl-CoA and malonyl-CoA [69]. Existing evidence has established linkages connecting *FASN* expression levels and activity with IMF deposition in swine. For instance, obese Wujin pigs were found to have increased *FASN* enzyme activity and mRNA abundance compared with lean Landrace pigs [70]. The *FASN* expression level was identified as an indicator of muscle fat accumulation in Italian Duroc pigs [71]. In the LDM tissue of the Diannan Small-ear and Tibetan pig (DSP-TP) group, the expression of *FASN* was roughly 10 times higher compared with the Landrace and Yorkshire (LL-YY) pig group, suggesting that the Chinese local pigs had higher lipid accumulation [72]. A clear overexpression of *FASN* was found in Iberian pigs with elevated IMF content in the LDM [73]. These investigations showed that *FASN* was a potential gene controlling fat characteristics in pigs and was associated with IMF content. Our findings support the earlier studies and reaffirm that *FASN* serves as a crucial regulator of IMF accumulation in pig PMM.

Interestingly, our GSEA analysis indicated that all four potential genes were significantly enriched in the pathways related to amino acid metabolism-related pathways (biosynthesis of amino acids and nitrogen metabolism), and both *DGAT2* and *FASN* were enriched in fat-metabolism-related pathways (fatty acid degradation and the PPAR signaling pathway). A study revealed a marked positive relationship between proline levels and the tenderness, juiciness, and overall acceptability of meat in Korean beef cattle [74]. The PPAR signaling pathway has been extensively documented to exert central regulatory functions in mediating fatty acid metabolism and meat quality development across mammalian species. The genes involved in this pathway mainly participate in the processes of lipid synthesis, fatty acid transport, and fatty acid oxidation, which have been reported to be closely related to the deposition of IMF [75]. In summary, we speculate that the differential expression of these potential genes could contribute to IMF deposition and meat quality modulation by mediating these signaling pathways.

In addition, we also predicted some miRNAs and lncRNAs associated with the potential IMF-related genes and constructed an mRNA (gene)–miRNA–lncRNA regulation network to further elucidate the function of and interconnections between the genes and non-coding RNAs (ncRNAs). Through the analysis of mRNA–miRNA interactions, we identified that *FASN* interacted with various miRNAs related to lipid metabolism, such as miR-103a-3p [76], miR-107 [77], and miR-485-5p [78]. Notably, the lncRNAs XIST, NEAT1, and KCNQ1OT1 were predicted to regulate *FASN* expression via the aforementioned miRNAs. Given that previous studies have highlighted the significant roles of XIST, NEAT1, and KCNQ1OT1 in lipid metabolism regulation [79,80,81], we hypothesize that this interaction network involving miR-103a-3p, miR-107, miR-485-5p, XIST, NEAT1, KCNQ1OT1, and *FASN* is crucial for IMF deposition in the PMM of pigs, which might provide novel insights for the regulation of pig fat deposition. Our findings identified genes associated with energy metabolism and lipid metabolism in PMM, and variations in the expression levels of these genes are likely to influence the IMF content in skeletal muscle. These insights could inform future strategies aimed at genetic improvement for meat quality.

While four specific genes related to IMF deposition were identified and validated, it is important to acknowledge some limitations within the present study. Firstly, the findings were derived from a bioinformatics approach utilizing a limited sample size in a single pig breed. Therefore, the expression levels of the genes require further validation through larger sample sizes and a broader range of pig breeds, employing more methodologies. Secondly, the specific roles of the identified genes and the targeted ncRNAs within the mRNA (gene)–miRNA–lncRNA regulation network in relation to IMF deposition require further functional validation through overexpression or knockdown experiments in cells or animal models.

## 5. Conclusions

In summary, we conducted a systematic analysis of transcriptome data pertinent to PMM IMF deposition in Saba pigs through an extensive bioinformatics approach. The results led to the identification of 20 hub genes and various pathways (such as the AMPK and PPAR signaling pathways and fatty acid metabolism) related to PMM IMF content in Saba pigs. Notably, the hub genes *DGAT2*, *PCK1*, *MELK*, and *FASN* might serve as potential genes specific to IMF deposition in pig PMM. The interaction network involving the miRNAs (miR-103a-3p, miR-107, and miR-485-5p), lncRNAs (XIST, NEAT1, and KCNQ1OT1), and *FASN* might be crucial for pig IMF deposition. These findings offer new perspectives on the molecular mechanisms underlying IMF deposition in pork and provide new molecular targets for genetic selection strategies to improve IMF content and meat quality in swine breeding. However, the precise functions of these genes in the regulation of porcine skeletal muscle fat metabolism warrant further investigation.

## Figures and Tables

**Table 1:** Sample information in this study.

Group	Number	Slaughter Age (d)	Live Weight Before Slaughter (kg)	IMF Content (%)
Low IMF	6	320	102.67 ± 6.54	4.23 ± 0.61 ^B^
High IMF	6	320	100.58 ± 11.10	5.65 ± 0.78 ^A^

Statistical comparisons between the low- and high-IMF group. The different capital letters denote significant differences at *p* < 0.01.

**Table 2:** Fatty acid composition (g/100 g FM) of PMM in the low- and high-IMF groups.

Components	Low IMF	High IMF	*p*-Value
C14:0	0.042 ± 0.005 ^b^	0.052 ± 0.007 ^a^	0.022
C16:0	0.938 ± 0.106 ^B^	1.778 ± 0.142 ^A^	0.008
C17:0	0.007 ± 0.003	0.010 ± 0.003	0.179
C18:0	0.593 ± 0.073	0.682 ± 0.083	0.078
C20:0	0.014 ± 0.001 ^b^	0.017 ± 0.002 ^a^	0.017
C23:0	0.001 ± 0.002	0.003 ± 0.002	0.069
C24:0	0	0.003 ± 0.005	0.177
C16:1	0.093 ± 0.013 ^B^	0.133 ± 0.026 ^A^	0.008
C18:1n9c	1.443 ± 0.188 ^B^	1.875 ± 0.260 ^A^	0.008
C20:1	0.035 ± 0.007	0.051 ± 0.017	0.071
C22:1n9	0.009 ± 0.005	0.006 ± 0.001	0.185
C24:1	0.001 ± 0.001	0.003 ± 0.002	0.076
C18:2n6c	0.293 ± 0.043	0.346 ± 0.081	0.183
C18:3n3	0.007 ± 0.001	0.008 ± 0.003	0.422
C20:2	0.011 ± 0.002 ^b^	0.015 ± 0.003 ^a^	0.047
C20:3n6	0.010 ± 0.001	0.011 ± 0.002	0.112
C20:3n3	0	0.001 ± 0.002	0.175
C20:4n6	0.061 ± 0.012	0.068 ± 0.012	0.318
ΣSFA	1.596 ± 0.179 ^b^	1.945 ± 0.233 ^a^	0.016
ΣUFA	1.963 ± 0.215 ^B^	2.516 ± 0.358 ^A^	0.009
ΣMUFA	1.581 ± 0.201 ^B^	2.067 ± 0.278 ^A^	0.007
ΣPUFA	0.382 ± 0.055	0.449 ± 0.091	0.149
Σω-3	0.007 ± 0.001	0.009 ± 0.004	0.241
Σω-6	0.364 ± 0.053	0.425 ± 0.085	0.160
Σω-6/Σω-3	54.517 ± 4.027	51.707 ± 13.412	0.641

Comparative analysis of fatty acid composition across low- and high-IMF pigs, with alphanumeric superscripts (capital/lower case) denoting significance at *p* < 0.01 and *p* < 0.05, respectively. ΣSFA, sum of saturated fatty acids; ΣUFA, sum of unsaturated fatty acids; ΣMUFA, sum of monounsaturated fatty acids; ΣPUFA, sum of polyunsaturated fatty acids.

**Table 3:** Amino acid composition (g/100 g FM) of PMM in the low- and high-IMF groups.

Components	Low IMF	High IMF	*p*-Value
Aspartic (Asp)	1.765 ± 0.076	1.750 ± 0.056	0.704
Threonine (Thr)	0.888 ± 0.033	0.887 ± 0.031	0.930
Serine (Ser)	0.698 ± 0.023	0.697 ± 0.022	0.900
Glutamine (Glu)	2.755 ± 0.119	2.732 ± 0.095	0.716
Glycine (Gly)	0.768 ± 0.029	0.773 ± 0.024	0.751
Alanine (Ala)	1.075 ± 0.035	1.073 ± 0.029	0.929
Cysteine (Cys)	0.185 ± 0.012	0.175 ± 0.019	0.299
Valine (Val)	0.973 ± 0.040	0.967 ± 0.026	0.738
Methionine (Met)	0.448 ± 0.031	0.438 ± 0.028	0.572
Isoleucine (Ile)	0.900 ± 0.043	0.888 ± 0.033	0.610
Leucine (Leu)	1.602 ± 0.063	1.602 ± 0.050	1.000
Tyrosine (Tyr)	0.605 ± 0.035	0.577 ± 0.029	0.161
Phenylalanine (Phe)	0.792 ± 0.026	0.787 ± 0.033	0.777
Lysine (Lys)	1.727 ± 0.063	1.722 ± 0.057	0.888
Histidine (His)	0.783 ± 0.053	0.758 ± 0.033	0.347
Argine (Arg)	1.190 ± 0.050	1.175 ± 0.037	0.568
Proline (Pro)	0.703 ± 0.020	0.723 ± 0.040	0.296
FAA	8.653 ± 0.324	8.635 ± 0.283	0.919
EAA	8.230 ± 0.335	8.178 ± 0.261	0.772
NEAA	10.528 ± 0.427	10.433 ± 0.308	0.668
TAA	17.858 ± 0.719	17.723 ± 0.526	0.720
EAA/TAA	0.461 ± 0.001	0.462 ± 0.002	0.573
EAA/NEAA	0.782 ± 0.003	0.784 ± 0.006	0.441

The amino acid composition was statistically compared between the low- and high-IMF groups. FAA, flavor amino acids; EAA, essential amino acids; NEAA, non-essential amino acids; TAA, total amino acids.

## Data Availability

The datasets PRJNA1223630 and GSE207279 for this study can be found in the NCBI Sequence Read Archive database and the GEO database, respectively. Furthermore, the data in this study will be made available on request.

## Supplementary Materials

The following supporting information can be downloaded at: https://www.mdpi.com/article/10.3390/ani15081181/s1, Table S1: Primer Sequences for qPCR Analysis. Table S2: The information on DEGs. Table S3: The results of the functional enrichment analysis of DEGs.
